# Vertebral growth modulation by posterior dynamic deformity correction device in skeletally immature patients with moderate adolescent idiopathic scoliosis

**DOI:** 10.1007/s43390-020-00189-z

**Published:** 2020-08-21

**Authors:** Yizhar Floman, Ron El-Hawary, Baron S. Lonner, Randal R. Betz, Uri Arnin

**Affiliations:** 1 Israel Spine Center, Assuta Hospital, 20 Habarzel, Tel Aviv, Israel; 2 Division of Orthopedic Surgery, IWK Health Center, University Ave, PO Box 9700, Halifax, NS 5850 B3K-6R8 Canada; 3 Mount Sinai Hospital, 1468 Madison Ave, New York, NY 10029 USA; 4 Institute for Spine and Scoliosis, 3100 Princeton Pike, Lawrenceville, NJ USA; 5 ApiFix LTD, Kochav Yokneam Bldg, 1 Hacarmel street, Yokneam Ilit, Israel

**Keywords:** Remodeling, Posterior, Dynamic, Correction, Idiopathic scoliosis

## Abstract

**Study design:**

Retrospective, comparative, multicenter.

**Introduction:**

Growth modulating spinal implants are used in the management of scoliosis such as anterior vertebral body tethering. A motion-sparing posterior device (PDDC) was recently approved for the treatment of moderate AIS. The purpose of this study was to determine if the PDDC can modulate growth in skeletally immature patients with AIS.

**Methods:**

From a database of patients treated with the PDDC over 4 years, we identified those who had a minimum of 2 years follow-up. Pre-operative and post-operative Cobb angles and coronal plane wedging of the apical vertebra were evaluated on standing full length radiographs. Independent sample *t* test and one-way ANOVA with post-hoc Tukey HSD analysis was used to compare three groups in varying skeletal maturity: Risser 0–1, Risser 2–3, and Risser 4–5.

**Results:**

45 patients (14.2-years old, 11–17) were evaluated with a mean pre-op curve of 46° (35°-66°). The average preoperative major curve magnitude, of either Lenke 1 or 5 curve type, was similar among the three groups 47.6°, 46° and 41.5°. Deformity correction was similar in the three groups, with reduction to 26.4°, 20.4° and 26.2°, respectively, at final follow-up [p < 0.05]. Pre-op wedging 7.4° (3.8°–15°) was reduced after surgery to 5.7° (1°–15°) (*p* < 0.05). Of those patients, Risser 0–1 (*n* = 16) had preoperative wedging of 9.5° (6°–14.5°) that was reduced to 5.4° (1°–8°) postoperatively (*p* < 0.05); Risser 2–3 (*n* = 15) had pre-op 7.7° (4°–15°) vs. post-op 7.0° (3°–15°); Risser 4–5 (*n* = 14) had pre-op 4.8° (3.8°–6.5°) vs. post-op 4.7° (3.7°–6.5°). Delta Wedging in Risser 0–1 stage was significantly different than for Risser 2–3 and for Risser 4–5.

**Conclusion:**

The posterior dynamic deformity correction device was able to modulate vertebral body wedging in skeletally immature patients with AIS. This was most evident in patients who were Risser 0–1. In contrast, curve correction was similar among the three groups. This finding lends support to the device’s ability to modulate growth.

## Introduction

Motion sparing and growth modulating spinal implants were recently incorporated into the surgical armamentarium of adolescent idiopathic scoliosis and currently utilize anterior implants such as vertebral body tethering (VBT) [1, 2] or vertebral body stapling (VBS) [3, 4]. Although VBT and to a lesser extent VBS, has been reported to promote growth modulation, their implantation necessitates an anterior approach to the spine. Many spine surgeons are less familiar with the anterior route and are more proficient with the posterior approach. A novel posterior dynamic deformity correction device (PDDC) (ApiFix Ltd., Misgav, Israel) was developed to provide a less invasive, motion sparing means to correct single Lenke 1 and 5 AIS curves and control the deformity over time [5–7]. The PDDC device has a ratchet mechanism that allows unidirectional elongation of an expandable rod which is made of titanium alloy with amorphous diamond-like ceramic coating. The expandable rod, with polyaxial rings (eye joint) at its extremity, is anchored to the spine with two pedicle screws that are implanted on the concavity of the main curve. The ratchet mechanism enables both immediate intra-operative curve reduction and potential gradual postoperative curve correction by unidirectional device elongation, which is driven by optional corrective spinal exercises performed by the patient. The device may be looked upon as an **internal brace** not dependent on brace wear compliance. This motion-sparing posterior device has a CE mark and was recently approved in the US by the Federal Drug Administration (FDA) for the treatment of moderate AIS. The ability of PDDC to significantly correct moderate AIS curves in patients fitting the current US indications i.e. 40°–60° single major curves reduced to ≤ 30° on bending, was recently reported [8]. It was found that in 82% of the cohort, it was possible to reduce the main curve to ≤ 30°at 2 years follow-up.

The purpose of this study was to determine if the PDDC can act as a true internal brace and modulate growth in skeletally immature patients with moderate AIS. If proved to significantly reduce the spinal curve and modulate vertebral wedging, it could enable future removal of the “internal brace” implant after skeletal maturity. Preliminary results have been accepted for presentation at the 2020 IMAST meeting [9].

## Materials and methods

From a registry of AIS patients treated with the PDDC (between 2015 and 2018), we identified those who had a minimum of 2 years of radiologic follow-up. As a marker for vertebral body growth modification induced by the altered scoliosis biomechanics, coronal plane wedging of the apical vertebra was evaluated. Two observers independently measured wedging of the apical vertebra on standing full length radiographs taken preoperatively and at final follow-up. The average of the two measurements was recorded. Independent sample *t*-test and one-way ANOVA with post-hoc Tukey HSD analysis was used to compare groups in varying skeletal maturation stages: Risser 0–1, Risser 2–3, and Risser 4–5.

We also recorded the preoperative and postoperative Cobb angles of the major instrumented curve at the last follow-up visit.

## Results

**Fig. 1:**
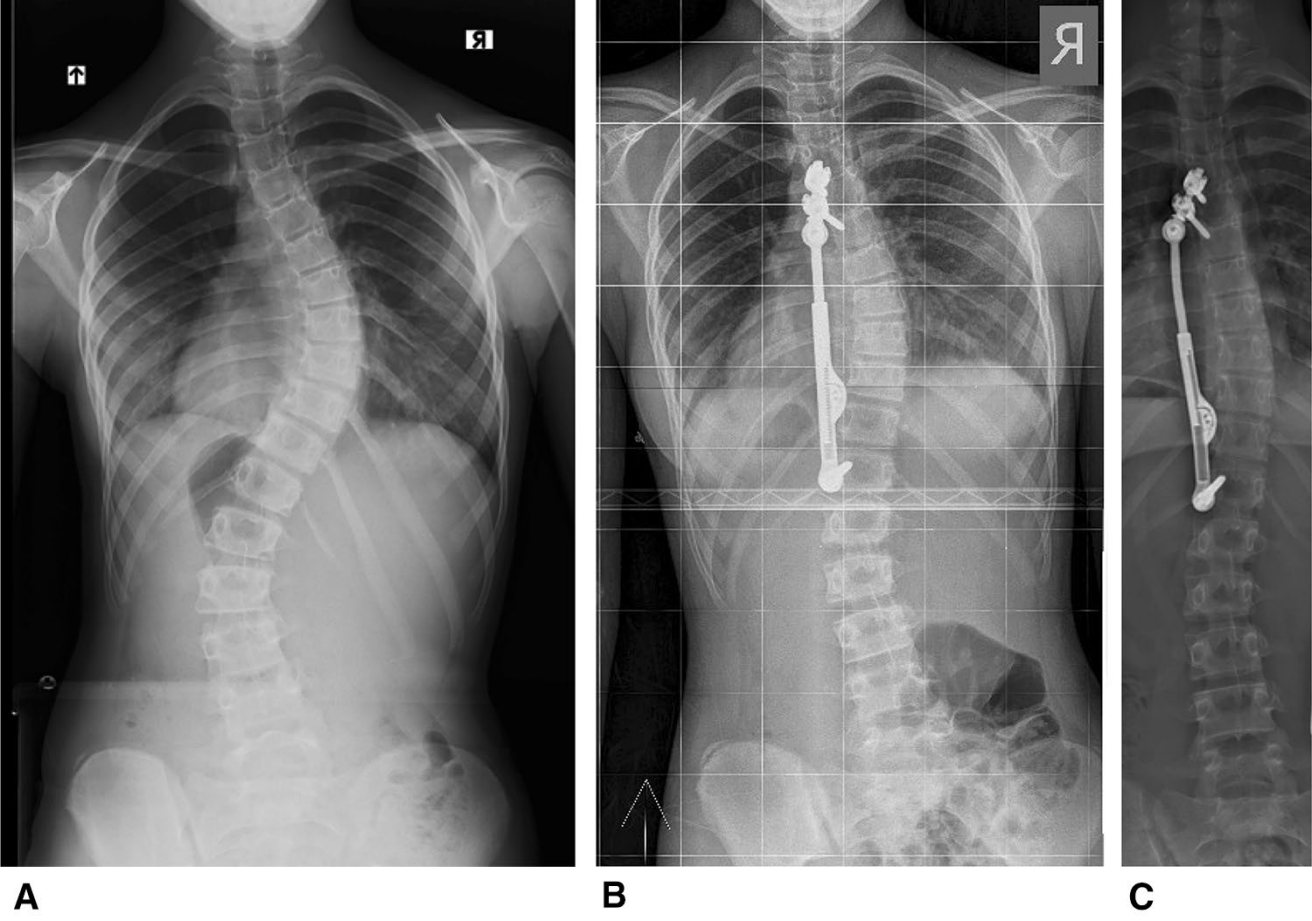
**a** 12-year old girl Lenke 1 58°, Risser grade 1. T9 coronal wedging is 10°. **b** Immediate postoperative x-ray. **c** At 2 years follow-up the curve measures 20° and coronal wedging is 4° (Risser 4 stage). In this case the device was anchored to the spine by two proximal pedicle screws via an “extender”

**Fig. 2:**
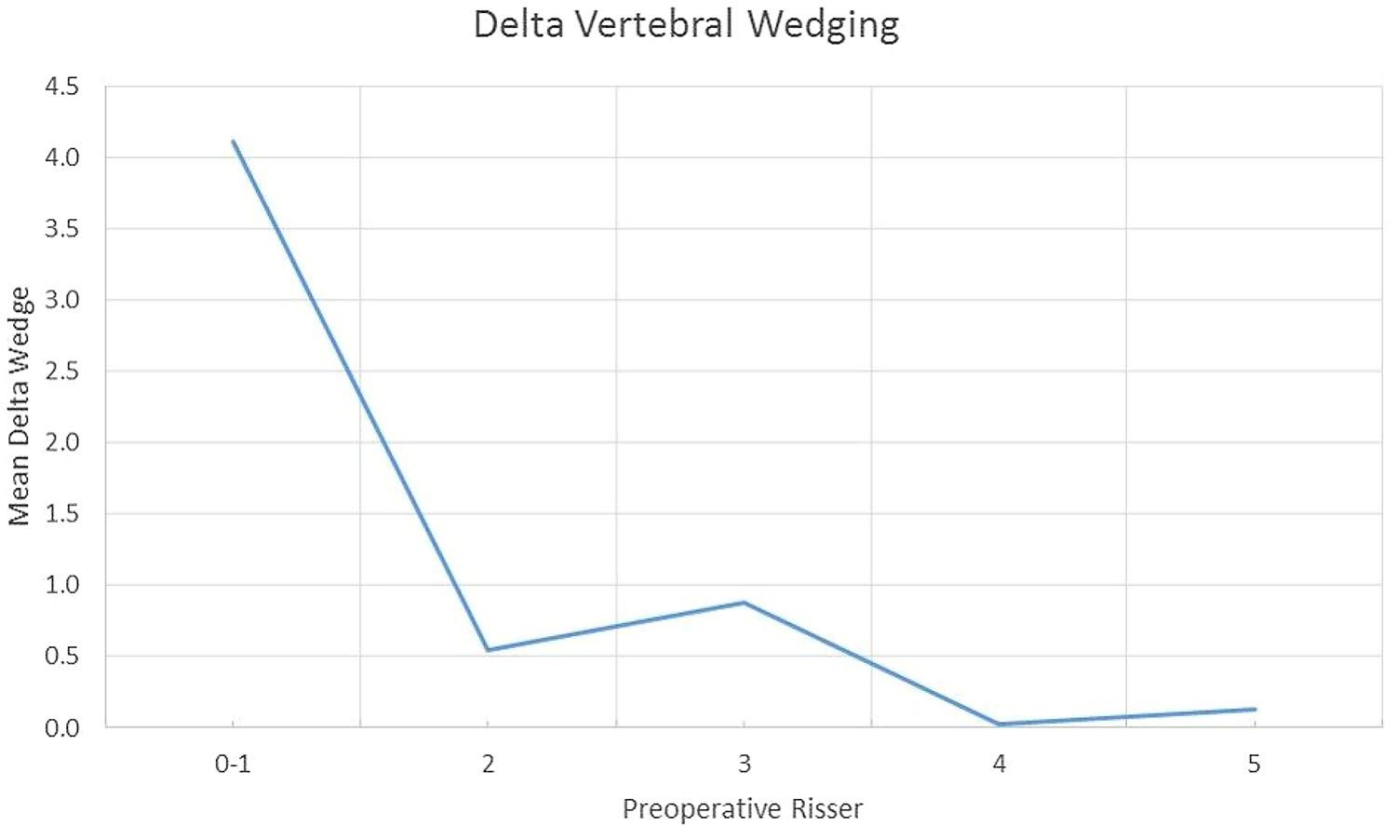
Delta coronal apical vertebra wedging between preoperative x-ray and last follow-up x-ray plotted against the preoperative Risser sign

From a registry of 220 patients that underwent PDDC surgery, 16 patients that were in a Risser 0–1 stage at the time of operation and had a minimum of 2 year follow-up were identified. In all 16 patients, the triradiate cartilage was closed at the time of surgery. This group was compared to 15 patients that that were in the Risser 2–3 stage and 14 that were in the Risser 4–5 stage at surgery and had a least 2 years of follow-up. The total number of patients evaluated was 45, of which 41 females and 4 males, aged 11–17 years (average 14.2). The indications for surgery were 40°–60° curves, reduced on lateral bending views to 35° or less with kyphosis not exceeding 55°. Non-compliant braced patients or patients with Lenke 5 curves with Cobb angles ≥ 35° were also subjected to PDDC surgery. There were 35 patients with Lenke type 1 curves and ten patients with Lenke type 5 curves (Fig. 1). Follow-up ranged from 2–4 years, average 2.3 years. All patients were Risser 4 or 5 at the end of the follow-up period. The cohort had a mean pre-operative major scoliosis curve of 46° (35°–66°). The average preoperative major curve magnitude, of both curve types, was similar among the three Risser groups 47.6°, 46° and 41.5°. Curves were reduced to 26.4°, 20.4° and 26.2°, respectively, at final follow-up [p < 0.05]. At last follow-up visit thoracic kyphosis increased on average by 7° in the Lenke 1 curves while lumbar lordosis decreased by 4° in the Lenke 5 curves. The average pre-operative coronal apical wedging of 7.4° (3.8°–15°) was reduced to 5.7° (1°–15°) at final follow-up (*p* < 0.05) (Table 1). Delta wedging for the entire cohort was 1.7° (− 0.5°–9°) (*p* < 0.05). Patients with Risser 0–1 grading (*n* = 16) had pre-operative wedging of 9.5° (6°–14.5°) compared to wedging of 5.4° (1°–8°) after surgery (*p* < 0.05); Risser 2–3 patients (*n* = 15) had pre-operative wedging of 7.7° (4°–15°) vs. post-operative wedging of 7.0° (3°–15°); Risser 4–5 patients (*n* = 14) had pre-operative wedging of 4.8° (3.8°–6.5°) versus post-operative wedging of 4.7° (3.7°–6.5°). The change in wedging before and after surgery was as follows: Risser 0–1 = 4.1° (0°–9°) (*p* < 0.05); Risser 2–3 = 0.7° (0°–4°), Risser 4–5 = 0.1° (− 0.5°–1.2°). Delta wedging in Risser 0–1 patients was significantly different from the delta value for Risser 2–3 and for Risser 4–5 (*p* < 0.05) (Figs. 1 and 2, Table 1).

**Table 1:** Apical wedging in degrees

	Preop. Apical wedging (range)	Apical wedging at last follow-up (range)	Delta wedging
All patients (45)	7.4° (3.8°–15°)	**5.7**°* (1°–15°)	**1.7**°*
Risser 0–1 (16)	9.5° (6°–14.5°)	**5.4**°* (1°–8°)	**4.1**°*
Risser 2–3 (15)	7.7° (4°–15°)	7.0° (3°–15°)	0.7°
Risser 4–5 (14)	4.8° (3.8°–6.5°)	4.7° (3.7°–6.5°)	0.1°

*denotes *p* < 0.05

Four patients underwent revision surgery: One case was converted to VBT as a result of partial screw pull-out. In the other three patients pedicle screw misplacement, nut loosening or ratchet malfunction were detected, revision surgery entailed re-instrumentation with the PDDC implant. None of the cases were converted to a fusion procedure.

## Discussion

We retrospectively compared the outcomes of three groups of patients with moderate AIS that were instrumented with PDDC, each group had a different Risser sign grade and was in a different stage of skeletal maturity (Risser 0–1, 2–3 and 4–5) at the time of surgery.

The three groups were compared in regards to the magnitude of main curve reduction and the extent of remodeling in the coronal plane wedging of the apical vertebra following surgery with PDDC. Average preoperative major curve magnitude, of either Lenke 1 or 5 types, was similar among the three groups 47.6°, 46° and 41.5°. Curves were reduced to 26.4°, 20.4° and 26.2°, respectively, at final follow-up (average 2.3 years). Sagittal plane alignment was largely maintained with only slight but clinically insignificant increase in thoracic kyphosis in the Lenke 1 curves by 7° and decrease in lumbar lordosis by 4° in the Lenke 5 curves.

The main goal of this retrospective review was to evaluate the ability of the PDDC implant to act as a “true” internal brace and modulate spinal growth in AIS patients. It was found that the PDDC device enabled significant vertebral growth modulation as well as curve correction when applied in individuals who were at the early stages of skeletally maturation i.e. Risser 0–1. The average pre-operative wedging of 9.5° in the latter group, was significantly reduced to 5.4° at final follow-up. The growth modulation achieved in this group was in accordance with the Hueter–Volkmann principle. In patients who were in more advanced stages pf skeletal maturity, PDDC growth modulation was negligible and insignificant (groups with Risser stages 2–3 or 4–5). In contrast, curve correction was similar among the three groups.

The Hueter–Volkmann principle describes the response of growing bones to applied compression forces [10]. Abnormal loading of the epiphyseal plate alters the “normal” growth pattern. In general, compression forces inhibit bone growth while distraction forces stimulate bone growth. For example, growth is retarded on the concave side of a scoliotic curve while on the convex side growth continues in a normal pace or is even stimulated. This asymmetrical spinal growth results in a wedge-shaped apical vertebra. The continuous asymmetric loading and asymmetric growth leads to curve progression resulting in a vicious cycle scenario [11].

The Hueter–Volkmann law has been implicated not only as the underling mechanism for scoliotic curve progression but also as the basis for bracing in scoliosis treatment. Braces are the main stay of non-operative treatment of progressing AIS curves in skeletally immature individuals. Application of a spinal brace in scoliotic individuals is an attempt to normalize physiologic mechanical loading conditions of the vertebral growth plate during the adolescent growth. It has been calculated, in a finite element model simulating a 26° Lenke 1 curve, that there is an increase stress at the growth plate by 37% between the concave and convex sides of the apical vertebra [12]. By changing asymmetric growth of a spinal curve during the growth spurt, bracing slows down or stops curve progression. This is what is called vertebral remodeling.

The magnitude of the applied forces and the time of force application by the spinal brace are crucial in inducing vertebral remodeling. In an animal model of a spinal deformity, it was found that full time force application created twice the effect of growth modulation achieved with part time application (24 h a day vs. 12 h) [13]. A similar time dependent effect was found in adolescents with idiopathic scoliosis wearing a brace. A dose dependent effect was found, greater hours of brace wearing correlated with less or no curve progression [14].

Bracing is not always successful. Among the many factors associated with failed bracing, poor brace wear compliance is a leading cause for failure. From analysis of experimental and clinical data it is obvious that full compliance with the bracing regimen is crucial. An external brace is almost never worn for 24 h a day. The PDDC, an “internal brace” acts continuously, 24 h a day seven days a week. Thus, application of sustained mechanical forces will normalize asymmetric stress distribution and normalize vertebral growth with vertebral remodeling and sustained correction of the deformity. The current study lends support to the assumption that the PDDC device acts as a growth sparing implant that eliminates asymmetric loading of the vertebral growth plate and provides for long term correction and stabilization of the curvature.

A similar biomechanical effect favoring vertebral remodeling occurs with VBT. Newton et al. [1] have demonstrated that tethering was able to alter vertebral growth and correct scoliosis at 2–4 years after surgery. These investigators stated that the indications for the procedure were AIS patients with flexible 45°–60° curves and Risser 0–1 [1]. The specified indications are almost identical to the indications for PDDC, AIS curves from 45°–60° demonstrating curve flexibility to 35° on lateral bending views. On the other hand, Murray et al. [15] reported that VBS failed to differentially influence vertebral body growth.

In a significant portion of braced AIS patients, curve progression occurs despite proper bracing. It was found that bracing, even with full compliance, in Risser 0–1 patients with curves exceeding 40°’, was associated with very high percentage of bracing failure between 70 and 100% [16]. Early intervention with the “internal brace” in such patients, before significant curve progression, would result in both immediate curve correction and better vertebral remodeling. Reducing the Cobb angle to lower values and smaller apical vertebral wedging by the PDDC device may allow future removal of the dynamic implant, after skeletal maturity, without the fear of recurrent curve progression.

The shortcomings of the current study are the small cohort, retrospective analysis, and the lumping together of Risser 0 and Risser 1 cases. An additional disadvantage of the current investigation is related to the measurement of the wedged apical vertebra. While the deformation of the apical wedge in scoliosis is three dimensional, we measured only the coronal wedge deformation. However, our measurements of apical vertebral wedging were similar to the measurements described in a finite element model of AIS [17]. Another aspect of curve morphology that was not evaluated in the present investigation was the shape of the intervertebral disc which is also affected by the altered mechanics in a spinal curvature.

In summary, this study provides support to the notion that the PDDC device can modulate growth in Risser 0–1 AIS patients. In these patients, the PDDC device was able to correct vertebral wedging, by reducing asymmetrical loading of the vertebral growth centers, in addition to providing immediate and post-operative curve correction. The growth and motion sparing ability of the PDDC instrumentation may allow to recruit the remaining vertebral growth to arrest and correct the spinal deformity.
